# VISCOSUPPLEMENTATION IN ANKLE OSTEOARTHRITIS: A SYSTEMATIC REVIEW

**DOI:** 10.1590/1413-785220162401139470

**Published:** 2016

**Authors:** Thiago Batista Faleiro, Renata da Silva Schulz, Jorge Eduardo de Schoucair Jambeiro, Antero Tavares, Fernando Moreira Delmonte, Gildásio de Cerqueira Daltro

**Affiliations:** 1. Hospital Universitário Edgard Santos, Salvador, BA, Brazil.; 2. Universidade Jorge Amado, Salvador, BA, Brazil.; 3. Hospital Santa Izabel, Salvador, BA, Brazil.; 4. Universidade Federal da Bahia, Faculdade de Medicina da Bahia, Salvador, BA, Brazil.

**Keywords:** Osteoarthritis, Viscosupplementation, Hyaluronic acid.

## Abstract

To evaluate the efficacy of viscosupplementation in patients with osteoarthritis of the ankle. A systematic review to evaluate the evidence in the literature on the use of viscosupplementation for osteoarthritis of the ankle. For this review, we considered blind randomized prospective studies involving the use of viscosupplementation for osteoarthritis of the ankle. A total of 1,961 articles were identified in various databases. After examining each of the articles, five articles were included in this review. Treatment with intraarticular hyaluronic acid is a safe treatment modality that significantly improves functional scores of patients, with no evidence of superiority in relation to other conservative treatments. Further clinical trials with larger numbers of patients are needed so that we can recommend its use and address unanswered questions***. Systematic Review of Randomized Clinical Trials.***

## INTRODUCTION

Osteoarthritis (OA) is the most prevalent rheumatic disease among people over 65 years of age.^1^ The disease can have an impact on different aspects of patients' life including social activities, relationships, body self-image and emotional well-being.^2^ ^,^ ^3^ According to the Center for Disease Control and Prevention of the United States, the current direct and indirect annual estimated cost for the treatment of OA is US$ 86.2 billion and in 2030 the disease will affect about 63 million Americans.^4^

Several factors can influence the onset and progression of OA, such as age, changes in metabolism, genetic and hormone factors, biomechanical changes and articular inflammatory processes.^1^ ^,^ ^5^

Primary osteoarthritis of the ankle is rare, most commonly secondary to fracture or ligament chronic instability.^6^ In recent years there has been, both in Brazil and worldwide, increased incidence of post-traumatic and inflammatory osteoarthritis of the ankle.^7^ When clinically evident, OA is characterized by joint pain, limitation of movement, crepitus, occasional effusion, and various degree of inflammation without systemic variables.^8^

The traditional conservative treatment for ankle OA includes simple analgesics, nonsteroidal anti-inflammatory drugs (NSAID), intra-articular corticosteroid injections, physical therapy, physical activity and weight reduction.^1^ ^,^ ^9^ New alternatives for surgical treatment have been developed, however, despite the improvement in ankle arthroplasty results, joint arthrodesis is still considered the gold standard for treatment in cases of failure of conservative treatment.^10^ Nevertheless, the burden on surrounding joints and the resulting sequelae, with consequent deterioration of the patient's functional quality after tibiotarsal arthrodesis, support the search for alternative therapies.^11^

The concept of viscosupplementation was developed in the 60s by Bazals et al.,^12^ but only by the end of the 80s it has been used for treatment of knee OA in Italy and Japan.^13^ Hyaluronic acid has both viscous and elastic properties. The degree to which each feature predominates depends on load conditions. This allows the synovial fluid the unique ability to function differently depending on the amount of shear force applied.^14^ Although widely used for the treatment of knee osteoarthritis, evidence for its usefulness in ankle osteoarthritis is limited.^15^ The purpose of the present review was to assess whether there is data in the literature to support the indication of viscosupplementation for the conservative treatment of ankle osteoarthritis.

## METHODS

Prospective randomized blind trials involving the use of viscosupplementation for ankle osteoarthritis were considered for this systematic review.

Studies involving patients of all ages and both genders, with tibiotalar joint osteoarthritis (radiologically and clinically defined) were included and the outcomes pain and functional performance were evaluated.

The searches for articles were conducted in the following electronic databases: Medline (1966 to May 20, 2014); Embase (1988 to May 20, 2014), Cochrane Database of Systematic Reviews (1988 to May 20, 2014). The descriptors used were: "ankle", "osteoarthritis", "viscosupplementation" and "hyaluronic acid".

The references of the selected studies were also analyzed in the search for papers that could have been lost in the electronic search. 

To minimize errors and reduce the potential bias, the search was conducted independently by two researchers. Disagreements were resolved by group discussion between authors.

The selection was made through the title and abstract to identify potentially relevant articles for analysis. When the title, keywords, and abstract proved insufficient information to determine their suitability for inclusion, analysis of full articles was carried out.

To evaluate the internal quality of the papers we used the criteria described by Jadad et al.^16^ Such analysis considers randomization, blinding of participants and loss of follow-up or exclusions. The maximum score is five points and a study is considered poor if it receives a score equal to or lower than three.

## RESULTS

**Figure 1:**
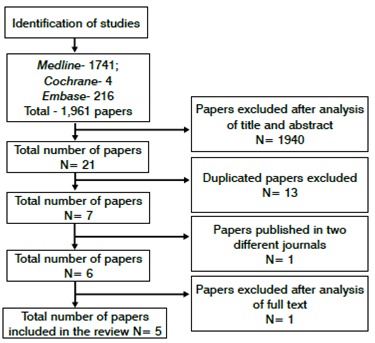
Research flow chart.

A total of 1,961 articles in different databases were identified. Analysis of the title and abstracts allowed the exclusion of 1,940 articles for not being prospective, randomized and blinded studies. Another 13 papers were excluded due to duplication. Therefore, seven articles remained with appropriate methodology for inclusion in the systematic review. However, a study from Salk et al.^17^ ^,^ ^18^ was published in two different journals and only one paper that presented a better description of the methodology was, then, included.^17^ The analysis of the articles full text led to the exclusion of another article due to lack of groups randomization. Therefore, five articles were included in the review, with a total population of 170 patients. (Figure 1)

Of the five articles evaluated, three of them compared the use of viscosupplementation with control using saline.^17^ ^,^ ^19^ ^,^ ^20^ The fourth study compared four different viscosupplementation regimens (single dose of 1, 2 or 3ml of hyaluronic acid and a fourth group with a 1ml/week for three weeks). The fifth study evaluated the use of hyaluronic acid with exercise therapy.^22^

The characteristics of the selected studies are shown in Chart 1.

**Chart 1:** Characteristics of the studies included in the systematic review.

Authors	Year of publication	Place of study	Intervention	Number of patients	Jadad et al.^16^ score
Cohen et al.^20^	2008	Canada	HA x Saline	30	3
Salk et al.^17^	2006	USA	HA x Saline	20	3
DeGroot et al.^19^	2012	USA	HA x Saline	64	4
Karatosun et al.^22^	2008	Turkey	HA x Physical therapy	30	3
Witteveen et al.^21^	2010	The Netherlands	4 HA regimens	26	3

HA: Hialuronic acid.

The technique employed to carry out the infiltration varied according to the literature. The anteromedial approach was described by Salk et al.^17^ and Witteveen et al.,^21^ preceded by local subcutaneous anesthesia. Karatosun et al.^22^ and Cohen et al.^20^ do not report whether the route used was anteromedial or lateral, while DeGroot et al.^19^ reported using them both, without, however, defining the criteria used for choosing between the different techniques or whether there were different patient outcomes. DeGroot^19^ and Cohen et al.^20^ reported the use of fluoroscopy.

The results of the studies that used saline as control evaluated different clinical outcomes. Cohen et al.^20^ chose as the primary outcome the Ankle Osteoarthritis Score (AOS) in four periods (second and sixth weeks and three and six months). Improvement was reported in both groups, greater in all evaluations of patients undergoing viscosupplementation, but with statistically significant difference only in the third month. DeGroot et al.^19^ used the American Orthopaedic Foot and Ankle Score (AOFAS) with primary outcome and AOS as secondary at the sixth and twelfth weeks. In patients receiving viscosupplementation there was improvement on both scores compared to the pre-treatment period, but only AOFAS was statistically significant. In the control group there was significant improvement only in the twelfth week, considering the AOS score. There was no significant difference between the groups in any of the evaluations. Salk et al.^17^ used the analysis of AOS score at three and six months as a primary endpoint. In both groups there was an improvement compared to baseline, but with no significant difference between them.

Karatosun et al.^22^ compared the intra-articular hyaluronic acid with exercise-based therapy. In both groups improvement in AOFAS Ankle - Hindfoot Score was identified at 12 months of follow-up, but with no statistically significant difference between groups.

Witteveen et al.^21^ attempted to determine the best viscosupplementation scheme comparing the use of 1, 2 or 3ml in one single dose or weekly doses of 1 ml for three weeks. They concluded that there was an improvement in the four groups, with better results in the group submitted to three infiltrations.

All studies reported low complication rate, none of them was considered serious.

## DISCUSSION

The viscosupplementation with intra-articular hyaluronic acid was approved in the US in 1997 and the American College of Rheumatology guidelines contemplate its use for the treatment of knee osteoarthritis since the year 2000.^23^ ^,^ ^24^ In 2008 the Osteoarthritis Research Society International included articular hyaluronic acid therapy as a modality with extended benefits for the treatment of patients with knee and hip osteoarthritis.^25^

Despite over a decade of clinical use, the literature still reports few studies on its use in the ankle joint. After extensive search we found only five articles with level 1 evidence, and four of these had low internal quality. The low level of evidence of studies on viscosupplementation is also observed in publications about foot and ankle surgery on other topics; there has not been over the past decade significant improvement in the studies' quality.^26^

Several techniques can be employed to increase the accuracy of infiltration such as ultrasound (US), fluoroscopy, and computed tomography (CT).^27^ ^,^ ^28^ Nevertheless, the relationship between greater effectiveness of the infiltration procedure and better clinical outcomes require further studies.^29^ In the articles selected for this study, we observed that all authors have chosen the anterior approach and two of them used fluoroscopy. We have not found evidence in the literature that fluoroscopy provides benefits to patients undergoing viscosupplementation in the ankle; this issue remains to be addressed in the future.

The initial objective of the authors was to perform a meta-analysis of selected works. But due to heterogeneity in study design it was not possible to perform statistical analysis.

Current evidence suggests that viscosupplementation for ankle osteoarthritis treatment is a safe and effective method, but without proven clinical superiority compared to other conservative treatment measures. Moreover, there is no data indicating which groups of patients benefit from this therapy, which is the best treatment regimen, the best technique to perform the procedure and the role of imaging techniques (US, CT, fluoroscopy).

## FINAL CONSIDERATIONS

Treatment with intra-articular hyaluronic acid is a safe therapeutic modality, which promotes a significant improvement of patients' functional scores, with no evidence of superiority over other conservative treatment measures.

New clinical trials with larger number of patients are needed prior to its recommendation, in order to answer remaining open questions.
